# Thymus-like phenotype in benign lymphoepithelial neoplasms of salivary glands: clinicopathological and molecular characterization and reappraisal of relationship to non-sebaceous lymphadenoma

**DOI:** 10.1007/s00428-025-04234-y

**Published:** 2025-09-06

**Authors:** Abbas Agaimy, Jan Laco, Christoph Schubart, Robert Stoehr, Lars Tögel, Stephan Ihrler

**Affiliations:** 1 Institute of Pathology, Erlangen University Hospital, Friedrich Alexander University of Erlangen-Nuremberg, Krankenhausstrasse 8-10, 91054 Erlangen, Germany; 2 Comprehensive Cancer Center, European Metropolitan Area Erlangen-Nuremberg (CCC ER-EMN), Erlangen, Germany; 3 The Fingerland Department of Pathology, Charles University Faculty of Medicine in Hradec Kralove and University Hospital Hradec Kralove, Hradec Kralove, Czech Republic; 4 DERMPATH Muenchen, Munich, Germany

**Keywords:** WHO classification, Lymphadenoma, Thymoma, EBV, Thymic carcinoma, Molecular profiling, Next-generation sequencing

## Abstract

Benign lymphoepithelial tumors of salivary glands had been restricted to sebaceous and non-sebaceous (NSLA) lymphadenomas. However, salivary neoplasms recapitulating carcinoma showing thymus-like elements (CASTLE) have been the subject of recent case reports. We reviewed clinicopathological, immunohistochemical, and molecular findings in 20 salivary gland tumors with thymus-like phenotype (18 histologically benign and two with malignant component). Original diagnoses were NSLA (*n* = 11) and unclassified thymus-like lymphoepithelial neoplasms (*n* = 9). Patients were 13 males and 7 females aged 28 to 83 years (median, 61). All tumors originated in the parotid with a median tumor size of 2.7 cm. A cystic component was noted in eight cases (40%). Histologically, the tumors were composed of large squamoid cells with indistinct cell borders, forming large irregular branching and anastomosing aggregates within lymphoid stroma with Hassall corpuscle-like structures and intraepithelial sprinkling of lymphocytes. All tumors were diffusely positive for p63/p40 and CK5/CK14. CD5 and CD117 were expressed in 13/20 (65%) and 15/19 (79%) cases, respectively. The malignant component in two cases showed lower CD5/CD117 expression. Targeted DNA sequencing revealed pathogenic/likely pathogenic *CYLD* mutations in 4/7 cases (57%). One case each had a mutation in *TAF1* + *WISP3*,* DNMT3A*, and *BCOR*. Targeted RNA sequencing revealed a *YAP1::MAML2* fusion in 1/7 cases. This is the first systematic study addressing the concept of thymus-like phenotype in benign lymphoepithelial salivary gland tumors, showing that the majority of NSLAs (65%) belong to this poorly characterized category. Frequent *CYLD* mutations in these histologically distinct tumors represent a novel addition to the spectrum of *CYLD*-mutated salivary neoplasms.

## Introduction

Salivary gland lesions composed of intimate admixture of epithelial cells and lymphoid tissue (benign and/or malignant) are collectively common. Their spectrum spans reactive inflammatory lesions (cystic and non-cystic lymphoepithelial sialadenitis in the context of Sjögren disease and HIV infection) [1, 2], benign neoplastic lesions (sebaceous and non-sebaceous lymphadenoma) [3, 4], low-grade malignant neoplasms (acinic cell and other low-grade carcinomas with prominent lymphoid stroma) [5, 6], high-grade malignancies (lymphoepithelial carcinoma) [7, 8], and intraparotid lymphomas with prominent epithelial inclusions/proliferations [9]. While the majority of these represent clearly defined and well-established entities, that have overstood the test of time, the rare non-sebaceous lymphadenomas (NSLA) remained an ambiguous entity with a highly heterogeneous morphological spectrum [3, 4]. In a prior study, our group had reported the second largest series of NSLA comprising nine cases, thereby delineating its probable origin from intranodal salivary inclusions (evidenced by the presence of hilum-like structures and of marginal sinus, lined by D2-40-positive endothelial cells), at same time highlighting a remarkable cytoarchitectural diversity of its epithelial component and frequent confusion with malignancies [4]. Notably, NSLA may display a variably cystic component or be totally solid [3, 4]. Their epithelial component varies from solid basaloid aggregates, indistinguishable from basal cell adenomas, to clear-cut duct formation, and to proliferating, larger, irregularly shaped and variably interconnected epithelial islands, associated with squamoid features, sprinkling of mature lymphocytes, and prominent lymphoid stroma in the background [3, 4]. The term “lymphoepithelial-type NSLA” has been applied to the latter pattern [4].

Based on own observations of several parotid gland tumors, histomorphologically overlapping with NSLA, at the same time showing variable atypical features, suggestive of a thymus-like phenotype, we conducted the current study to 1) document our experience with salivary gland neoplasms showing thymus-type epithelial features that we have collected prospectively, 2) test the expression status of CD117 and CD5 as two markers characteristic of thymic epithelial differentiation in these tumors, 3) address the notion that these might be overlapping with lesions in the spectrum of NSLA, and 4) perform molecular profiling to identify any recurrent genetic drivers in them, using both DNA-based and RNA-based targeted next-generation sequencing approaches.

## Material and methods

The 26 cases were retrieved from our routine and consultation files. Seventeen archival cases coded as NSLA (9 of them had been included in a previous study [4]) have been critically reevaluated by two authors (AA and SI) and subtyped into either genuine NSLA (defined as biphasic encapsualted lymphoepithelial nodule with its epithelial component clearly likened to benign adenomas of the salivary glands, i.e., basaloid solid aggregates, variable duct-like structures, or biphasic glands) or as thymus-like lesions (defined as lymphoepithelial-like growth of larger, squamoid pale eosinophilic cells with syncytial growth pattern and variable Hassall-like corpuscles, and/or abrupt keratin foci, closely mimicking normal thymus tissue, lacking duct formation, and/or bilayering with sprinkling of intraepithelial lymphocytes). In addition, nine prospectively collected tumors with thymus-like phenotype are included. The presence or absence of cystic component and of frankly malignant features was recorded. Because most cases were seen in the context of second opinion, detailed follow-up data were not available.

### Immunohistochemistry

Due to the consultation nature of most cases and the variable tissue availability, immunohistochemistry (IHC) was performed in different laboratories and with different numbers of antibodies (p63/p40, CK5/14, p16, TP53). CD5 (clone SP19, 1:50, Zytomed) and CD117 (clone EP10, 1:100, Quartett) have been centrally stained using a Ventana BenchMark Ultra autostainer (Ventana, Roche, Basel, Switzerland) according to the manufacturer’s instructions.

Epstein Barr virus (EBV) in-situ hybridization (EBER 1/2 probes, ZytoVision, Bremerhaven, Germany) was performed on a subset of cases according to the manufacturer guidelines. Positive and negative controls were used throughout.

HPV status was tested in case 13 using a AmoyDx® human papillomavirus (HPV) genotyping detection kit which is a real-time PCR assay enabling qualitative detection of up to 19 high-risk HPV DNA (HPV 16, 18, 26, 31, 33, 35, 39, 45, 51, 52, 53, 56, 58, 59, 66, 68, 70, 73, and 82) and 2 low-risk HPV DNA (HPV 6 and 11). Case 12 and 15 were tested using the methods described previously [10].

### Next-generation sequencing

A subcohort (*n* = 8) of thymus-type neoplasms was subjected to targeted RNA-based and/or DNA-based next-generation sequencing on tumor RNA and DNA, extracted from formalin-fixed paraffin-embedded (FFPE) tissue sections using standard protocols. Molecular analysis for DNA sequence variants was performed on seven tumor samples using the TruSight Oncology Panel (TSO500 DNA Panel, Illumina, Inc., San Diego, CA, USA), which includes 523 cancer-associated genes. Six DNA-tested tumors were in addition subjected to RNA-based targeted NGS using either the RNA component of the TSO500 panel (four cases) or the TruSight RNA Fusion panel that encompasses 507 fusion-related genes (two cases). Due to material limitation, one additional tumor was subjected only to RNA-based targeted NGS using the TruSight RNA Fusion panel. All tests were performed according to the manufacturer’s protocol.

## Results

### Distribution of the subcohorts after critical reevaluation of archival diagnoses

Of the 17 archival NSLA cases, 6 have been confirmed as NSLA and 11 as lymphoepithelial tumors showing thymus-like morphology. Together with 9 prospectively collected thymus-like lymphoepithelial neoplasms, the final cohort comprised 6 genuine NSLA and 20 lymphoepithelial neoplasms with thymus-like phenotype, the latter including two cases with transition to a histologically malignant carcinomatous component. Major clinicopathological, immunophenotypic, and molecular features of the different subcohorts are presented below.

### Clinical findings in genuine non-sebaceous lymphadenomas (NSLAs; *n* = 6)

The six NSLAs affected four males and two females aged 35 to 84 (median, 59) (Table 1). All tumors originated in the parotid gland. The tumor size ranged from 0.6 to 1.7 cm (median, 1.4).

**Table 1:** Clinicopathological features of genuine non-sebaceous lymphadenomas (NSLA; *n* = 6)

No	Age/sex	Size (cm)	Cystic component	Histological pattern	Hassall-like bodies	p63/p40	CK5/CK14	CD5	CD117
1	62/M	1.5	+	NSLA-basaloid	Absent	+	+ basal	-	-
2	59/F	1.3	+	NSLA-basaloid	Absent	+	+ basal	-	NA
3	35/M	1.7	+	NSLA-basaloid	Absent	+	+ basal	-	-
4	49/F	1.4	+	NSLA-basaloid	Absent	+	+ basal	-	-
5	60/M	0.6	No	NSLA-basaloid	Absent	+	+ basal	-	NA
6	84/M	1.7	No	NSLA-basaloid	Absent	+	+ basal	-	+ (luminal)

### Histopathological and immunohistochemical findings in genuine NSLAs

**Fig. 1:**
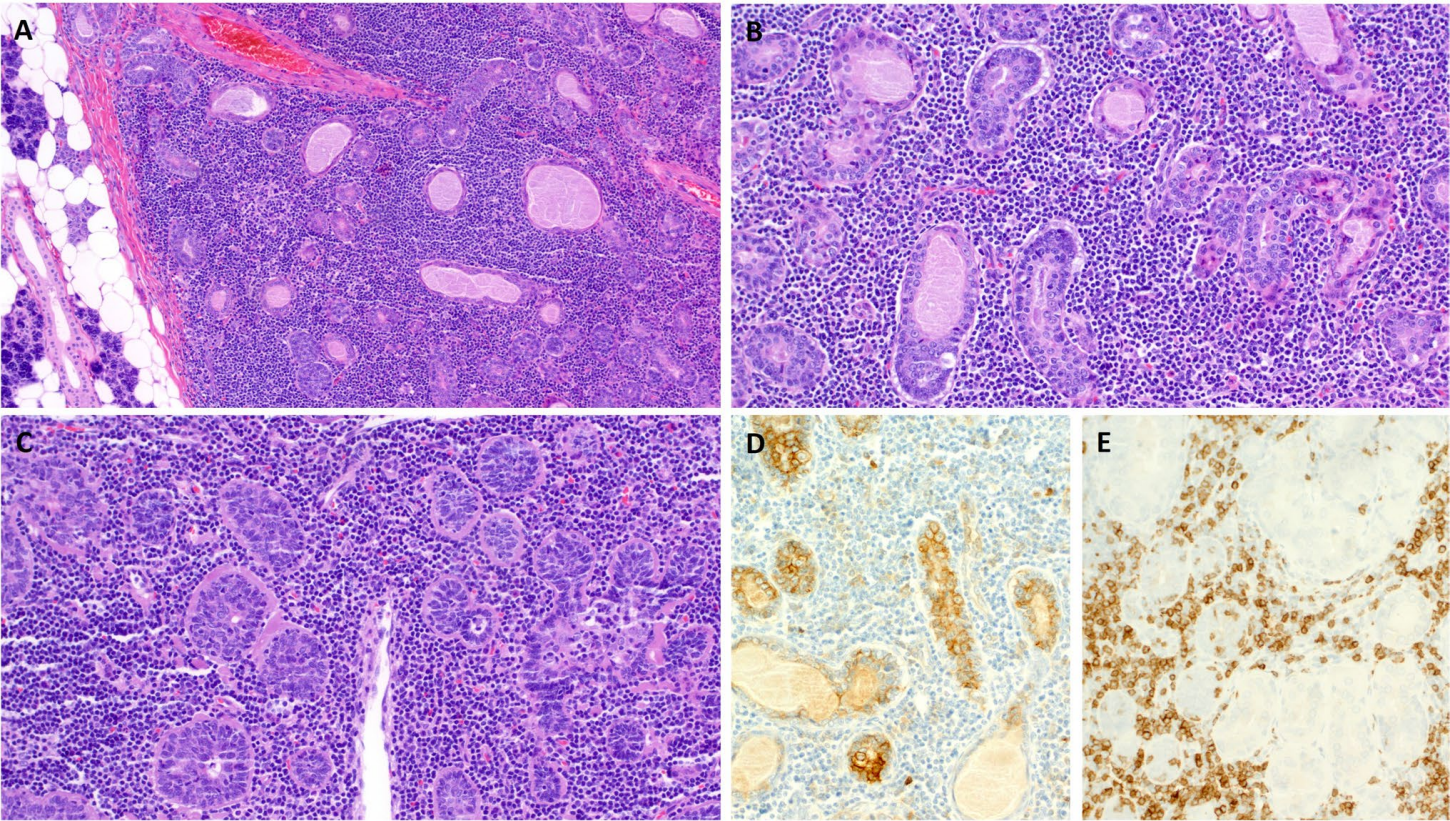
Genuine non-sebaceous lymphadenomas were encapsulated (**A**) and showed varied admixture of salivary-type tubules (**B**) and solid basaloid aggregates surrounded by basement membrane-type hyaline material (**C**). They expressed CD117 in a luminal/ductal pattern (**D**) and were all negative for CD5 (**E**; note prominent staining of T cells)

Histologically, the six tumors were characterized by scattered basaloid salivary-type epithelial nests and aggregates with variable ductal formation and pericellular basement membrane-like material (Fig. 1A–C). Intraepithelial lymphocytosis was absent. Four of the six NSLAs (67%) had a variable cystic component.

By immunohistochemistry, all six tumors expressed p63/p40 and CK5/CK14 with basal/peripheral distribution pattern. CD117 was expressed luminally in one of four cases (Fig. 1D). CD5 was negative in all six cases (Fig. 1E).

### Clinical features of thymus-like lymphoepithelial salivary neoplasms (*n* = 20)

All tumors originated in the parotid gland. Patients were 13 males and 7 females aged 28 to 83 years (median, 61). None had a known autoimmune sialadenitis or other associated relevant diseases.

### Pathological findings in thymus-like lymphoepithelial salivary neoplasms (*n* = 20)

**Fig. 2:**
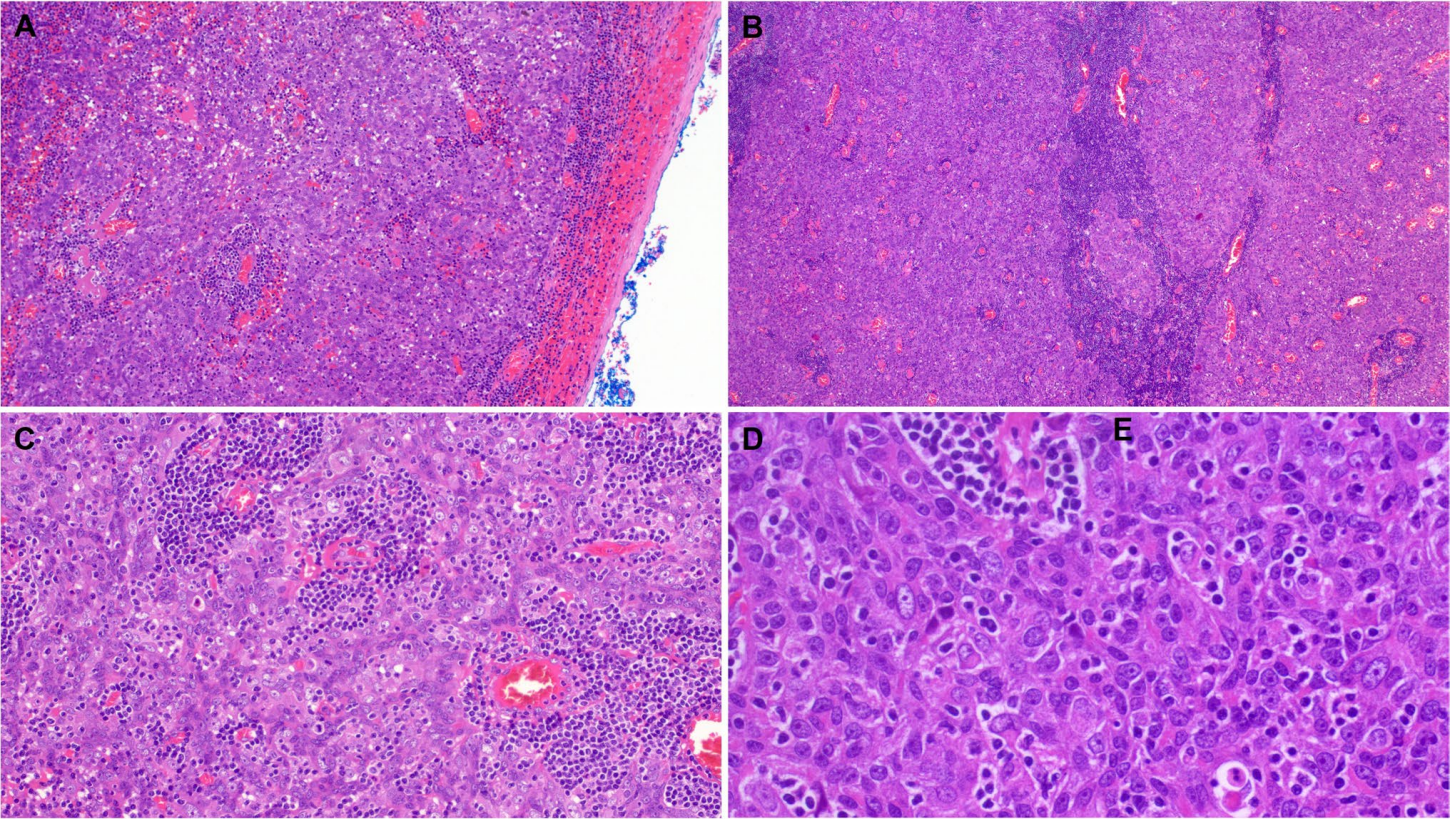
**A** Thymus-like lymphoepithelial neoplasms were well demarcated and encapsulated. **B, C** They are composed of prominent confluent irregularly branching aggregates of large pale eosinophilic squamoid cells within variable lymphoid stroma. Prominent lobulation is seen in cellular areas (**B**). **D** Higher magnification shows ovoid plumb epithelial cells reminiscent of epithelial thymoma cells with sprinkling of lymphocytes

**Fig. 3:**
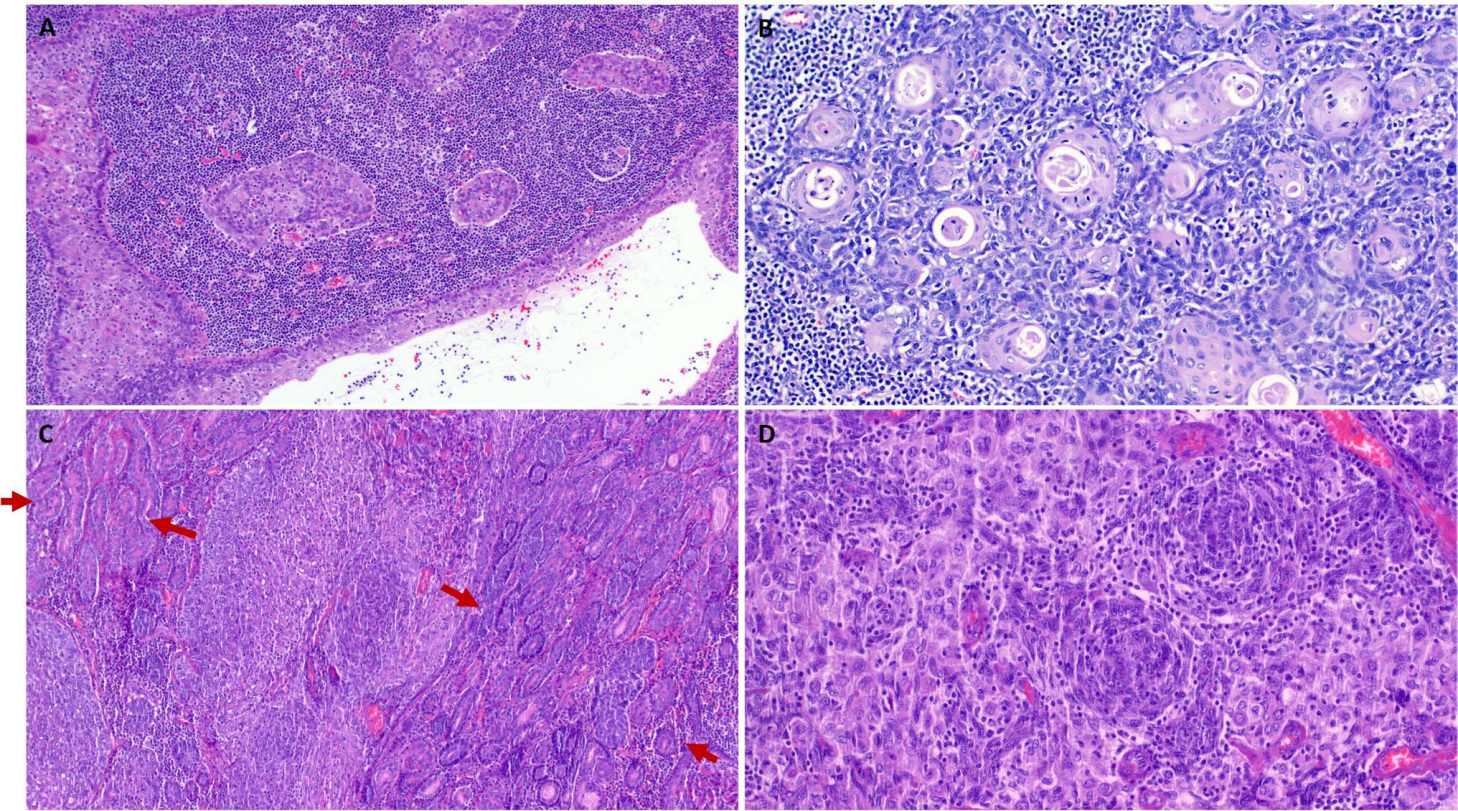
**A** This tumor showed prominent cystic component with similar squamoid epithelial lining as the remainder of the lesion. **B** Prominent Hassall body-like tubular structures with pale-stained luminal secretion. **C** This tumor showed pale-stained larger lobular aggregates and centrally located concentric whorls (midfield); note prominent residual NSLA component on the right and left field (arrows). **D** Higher magnification of the concentric whorls from same case

The tumor size ranged from 1 to 10 cm (median, 2.7). All tumors had a well-delineated periphery, delimited by a thin fibrous capsule without evidence of infiltrating growth or entrapment of native salivary lobules/acini (Fig. 1A). Histologically, 18 tumors were uniformly low grade, composed of large, pale-eosinophilic squamoid cells with indistinct cell borders, lacking classical squamous cell features, and forming large, irregularly shaped branching and anastomosing aggregates within prominent lymphoid stroma (Fig. 1B, C). The nuclei were ovoid to elongated and enlarged with vesicular chromatin and variably recognizable nucleoli (Fig. 1D). The degree of atypia was generally low to moderate. A cystic component was noted in eight cases (40%) and was predominant in one (Fig. 3A). Concentric squamoid aggregates closely mimicking thymic Hassall corpuscles were noted to a variable extent in all cases (Fig. 3B, D). Prominent sprinkling of mature lymphocytes within the tumor epithelium was seen in all cases (Figs. 2D, 3D). Additional foci of classical (basaloid and/or ductal) NSLA were seen in two cases (Fig. 3C; arrows). Frankly malignant features (malignant cytology, increased mitotic activity, coagulative necrosis) were present as a malignant component in two tumors (see below). However, nuclear enlargement was judged to be worrisome in most cases but was not enough to be classified as malignant.

### Immunohistochemical findings in thymus-like lymphoepithelial salivary neoplasms (*n* = 20; Table 2)

**Fig. 4:**
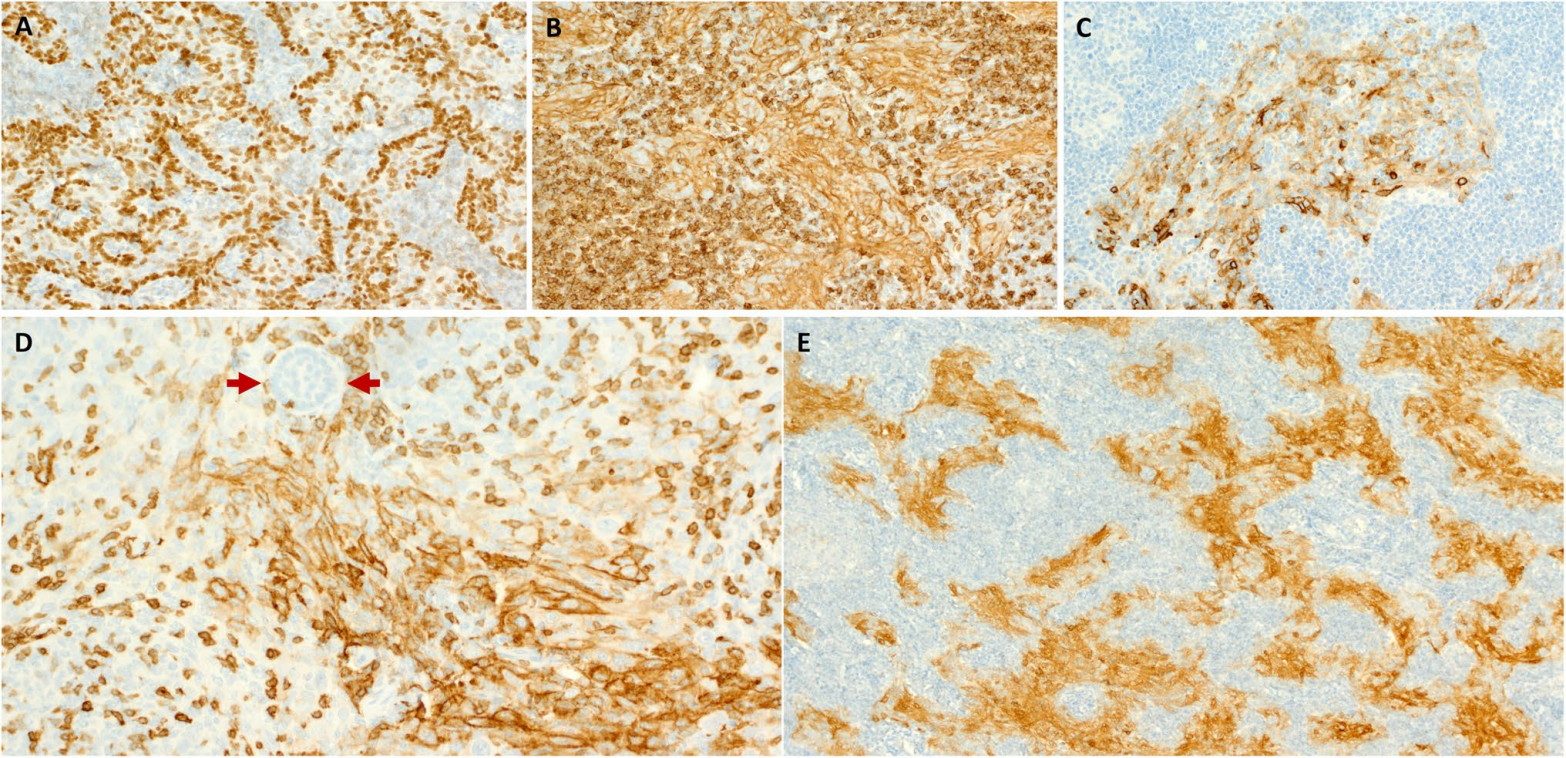
Representative images of immunohistochemical findings in thymus-like tumors. **A** Diffuse expression of p63/p40. **B** Diffuse expression of CD5. **C** CD117 reactivity. **D** Area with focal NSLA component showed lack of CD5 expression in the NSLA (upper midfield; arrows) compared to strong reactivity in the thymus-like area (lower midfield). **E** Diffuse expression of p16

All tumors showed diffuse expression of p63/p40 (Fig. 4A) and CK5/CK14 and lacked the zonal distribution into basal and luminal as noted in genuine NSLAs. CD5 (Fig. 4B) and CD117 (Fig. 4C) were expressed in 13/20 (65%) and 15/19 (79%) cases, respectively. In the tumors with residual NSLA component, CD5 was limited to the thymus-like areas (Fig. 4D; arrows). P16 was positive in seven of nine cases (diffuse in six and variable in one; Fig. 4E). All three p16-positive cases tested for oncogenic HPV were negative. All nine tumors tested for EBV by CISH assay (including the two cases with malignant component) were negative (Table 2).

**Table 2:** Clinicopathological features of thymus-like lymphoepithelial salivary gland tumors (*n* = 20)

No	Age/sex	Size (cm)	Cystic component	Original diagnosis	Hassall-like bodies	p63/p40	CK5/CK14	CD5	CD117	P16	HPV	EBER
1	51/M	2.3	+	NSLA	Present	+	+	−	NA	NA	NA	NA
2	62/M	2.8	No	NSLA	Present	+	+	−	+	NA	NA	NA
3	65/M	3.5	No	NSLA	Present	+	+	+	+	NA	NA	NA
4	50/M	1.4	+	NSLA	Present	+	+	−	−	NA	NA	NA
5	28/F	2.4	No	NSLA	Present	+	+	+	+	NA	NA	NA
6	53/F	2.5	+	NSLA	Present	+	+	−	+/−	NA	NA	NA
7	71/F	2	No	NSLA	Present	+	+	−	−	NA	NA	NA
8	51/M	2.6	No	NSLA	Present	+	+	−	−	NA	NA	NA
9	77/F	1.6	+	NSLA	Present	+	+	+	−	NA	NA	NA
10	78/M	1	No	NSLA	Present	+	+	+	+	NA	NA	NA
11	61/M	1	+	NSLA	Present	+	+	−	+	NA	NA	NA
12	51/M	4	Prominent	Unclassified thymus-like lymphoepithelial neoplasm	Present	+	+	+	+	+	−	−
13	65/F	2.9	No	Unclassified thymus-like lymphoepithelial neoplasm	Present	+	+	+	+	+	−	−
14	71/M	3.6	No	Unclassified thymus-like lymphoepithelial neoplasm	Present	+	+	+	+	+/−	NA	−
15	53/M	4	No	Unclassified thymus-like lymphoepithelial neoplasm	Present	+	+	+	+	+	−	−
16	80/F	4.5	+	Unclassified thymus-like lymphoepithelial neoplasm	Present	+	+	+	+	−	NA	−
17	81/M	3.8	No	Unclassified thymus-like lymphoepithelial neoplasm	Present	+	+	+	+	+	NA	−
18	56/M	NA	+	Unclassified thymus-like lymphoepithelial neoplasm	Present	+	+	+	+	+	Failed	−
19	48/F	10	No	Unclassified thymus-like lymphoepithelial neoplasm + malignant component	Present	+	+	+	+/−	−	NA	−
20	83/M	NA	No	Unclassified thymus-like lymphoepithelial neoplasm + malignant component	Present	+	+	+	+/−	−	NA	−

*NA* not available, *NSLA* non-sebaceous lymphadenoma

### Thymus-like salivary neoplasms with malignant transformation (*n* = 2)

**Fig. 5:**
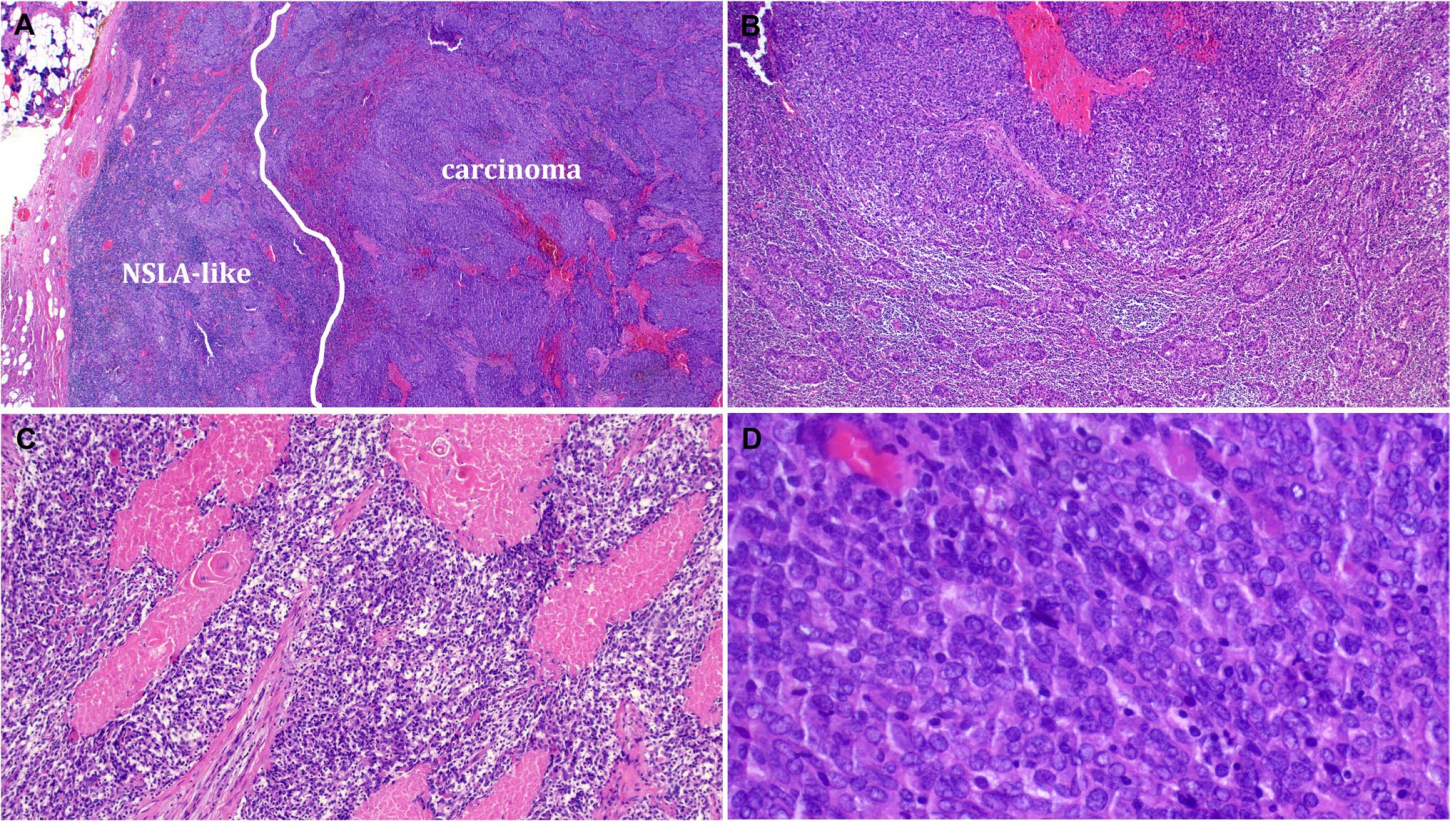
Example of malignant transformation in thymus-like neoplasms. **A** Encapsulated tumor showed transition from bland-looking lymphoepithelial tumor to highly cellular malignant component. **B** Higher magnification showing the transition from benign-looking (lower part) to cell-rich malignant component (upper part). **C** Multifocal tumor necrosis. **D** Higher magnification of the malignant component showing high cellularity with monotonous cytology and scattered lymphocytes

Two of the 20 thymus-like tumors showed transition from the bland-looking thymoma-like component to a large cell, poorly differentiated malignant component with diffuse solid growth of large epithelioid to ovoid cells with vesicular chromatin, prominent nucleoli, brisk mitotic activity, and multiple foci of necrosis. These frankly malignant areas were associated with progressive loss of thymus-like cytological and stromal characteristics including absence of prominent squamoid cell features, lack of Hassall-like corpuscles, and diminished intraepithelial lymphocytic sprinkling (Fig. 5A–D). The affected patients were a 48-year-old woman and an 83-year-old man. By immunohistochemistry, both tumors expressed diffusely squamous cell markers (p63/p40 and CK5/14) with variable expression of CD5 and CD117 (lost in the high-grade areas; not shown).

### Molecular findings in thymus-like lymphoepithelial salivary neoplasms (*n* = 8; Table 3)

Molecular testing was performed on a total of eight tumors. Seven thymus-like lymphoepithelial tumors were tested using the TruSight-500 DNA Panel (TSO500 DNA cancer panel, Illumina). In addition, targeted RNA sequencing was performed on seven cases using either the TruSight RNA Panel of Illumina (two cases) or the RNA part of the TSO500 Panel (five cases).

Remarkably, a pathogenic or likely pathogenic *CYLD* mutation was detected in four of seven cases using the TSO500 panel (57%). Two other cases had pathogenic mutations involving the *TAF1* and *WISP3* genes in one case and a *DNMT3A* mutation in the other case. One malignant case revealed a pathogenic *BCOR* variant in addition to an out-of-frame *NUP214::ABL1* fusion and low-level *FGFR1* amplification (copy number: 3).

*CYLD* mutations occurred in isolation in three cases and concurrent with an *NRAS* mutation in one case. Three of the detected *CYLD* mutations were terminating mutations resulting in protein truncation (Table 3). One tumor showed a splice-acceptor site mutation. They clustered between codon 485 and codon 947 of the *CYLD* gene. Their variant allele frequencies ranged from 2.7 to 21.7%. The splice-acceptor site mutation is likely to result in aberrant splicing and exon loss, causing a frameshift of the open reading frame of the gene, which in turn may lead to the generation of an alternative stop codon and loss of protein expression. Accordingly, the splice acceptor mutation detected in one case is predicted as very likely inactivating.

**Table 3:** Molecular findings in thymus-like lymphoepithelial salivary gland tumors (*n* = 8)

No	Histologically malignant component?	RNA Panel	TSO500DNA
1	-	*YAP1::MAML2*	NA
2	-	No fusion	***TAF1***: c.3670G > T (p.Glu1224Ter); VAF: 6,4% (likely pathogenic) ***WISP3***: c.648dup (p.Thr217AspfsTer); VAF: 13 51,5% (likely pathogenic)
3	-	No fusion	***CYLD***: c.2841C > A (p.Tyr947Ter); VAF: 18,2% (likely pathogenic)
4	-	No fusion	***CYLD***: c.1927G > T (p.Glu643Ter); VAF: 6.2% (pathogenic)
5	-	NA	***DNMT3A***: c.2204A > G (p.Tyr735Cys); VAF: 2,3% (likely pathogenic)
6	-	No fusion	***CYLD***: c.1453dup (p.Tyr485LeufsTer15); VAF: 21.7% (pathogenic) ***NRAS***: c.181C > G (p.Gln61Glu); VAF: 2.3% (pathogenic)
7	-	No fusion	***CYLD***: c.1519-2A > C Splice-Acceptor; VAF:2.7% (likely pathogenic)
8	Yes	*NUP214::ABL1* (out-of-frame)	***BCOR***: c.1024C > T (p.Arg342Ter); VAF: 81% (pathogenic) *FGFR1* (8p11.23) low-level amplification (CN: 3)

Targeted RNA sequencing revealed a *YAP1::MAML2* gene fusion in one of seven cases; the other five tumors had no detectable in-frame gene fusions. The fusion-positive tumor was predominantly cystic with no clear-cut malignant features and lacked features of metaplastic thymoma. Unfortunately, DNA testing was not available in this case and the *CYLD* gene is not among the included genes, so the persence or absence of a *CYLD* mutation cannot be addressed. An out-of-frame fusion was detected in one malignant case (Table 3).

## Discussion

Non-sebaceous lymphadenoma (NSLA) is a rare, benign, well-circumscribed lymphoepithelial tumor, first described by Auclair et al. in 1991 [11] and then recognized as a tumor entity by the WHO classification in 2005. In the current WHO classification 2022, NSLA and SLA are included under the heading of “lymphadenomas” [12]. Together, they represent 0.1% of all salivary gland neoplasms and < 0.5% of salivary adenomas [12]. A few case series have tried to delineate its histogenesis and at the same time to address the potential diagnostic confusion associated with this rare entity [3, 4]. Notably, the presence of a hilum-like structure and of marginal sinus lined by D2-40-positive endothelial cells and the nearly restricted occurrence in the parotid were considered strong evidence for derivation of NSLA from intranodal salivary inclusions, a concept not different from that of Warthin tumor [4]. However, the histogenetic origin and pathogenesis of these uncommon lesions remain ambiguous [13].

Over the last few years, we encountered several cases of salivary gland neoplasms that we could not classify exactly into any of the available categories of salivary neoplasia. We reported these lesions descriptively as *unclassified lymphoepithelial neoplasms showing thymus-like phenotype*. Notably, many of these tumors showed equivocal (borderline) cytological features that were judged at the time of primary reporting as being worrisome for malignancy, but lacked frankly malignant cytology, precluding a definitive carcinoma diagnosis. More recently, we encountered two tumors that showed transition to a frankly malignant histology. This prompted us to perform the current study to gain more insight into the nosology and pathogenesis of these rare and poorly understood ambiguous neoplasms of presumable thymus-like origin.

Our study shows that benign salivary lymphoepithelial neoplasms (restricted to the parotid gland) form a morphological-biological continuum with smaller lesions, historically called NSLA with lymphoepithelial pattern on the one end and frankly malignant tumors on the other end of a spectrum. In between, tumors showing intermediate features that fall short of being called carcinomas exist and are Likely more common than the truly malignant cases. These tumors pose several problems regarding 1) their relationship to NSLA, 2) their histogenesis, 3) their biological behavior, 4) their diagnostic immunomarkers (CD5 and CD117), and 5) their yet to be better delineated genetic landscape.

Regarding relationship to NSLA, our study showed that most NSLAs display thymus-like morphological and immunophenotypic features. Notably, genuine NSLA showing clear-cut salivary morphology with frequent duct formation and basaloid (basal cell adenoma-like) aggregates are uncommon, representing 35% of our archival NSLA cases.

Regarding their histogenesis, no convincing histological evidence exists that can explain the emergence of the thymus-like phenotype in these neoplasms. We have not encountered any thymic remnants associated with these lesions and no convincing evidence has been reported in the literature in the above-cited case reports [14–24]. In the thyroid gland, where intrathyroidal thymic neoplasms are generally accepted and included in the current WHO classification, origin from intrathyroidal thymic remnants or via metaplasia has been discussed [25, 26]. Indeed, the significant morphological overlap of our cases with NSLA and the observation of remnants of NSLA in some of our cases point to origin of these tumors from preexistent salivary inclusions and/or NSLA via metaplasia or transdifferentiation. Also, the nearly exclusive intraparotid occurrence of these tumors represents another argument for origin from intranodal salivary lesions, similar to Warthin tumors and genuine NSLA.

With regard to their biology, a classification scheme analogous to that of intrathyroidal thymic neoplasms might be justified [25, 26]. There, intrathyroidal thymoma and thymic carcinoma are distinguished, based on histological criteria defined for primary thymic neoplasms. However, more well-documented and clearly defined tumors with long-term follow-up are needed before a reliable conclusion can be drawn regarding the anticipated behavior. Notably, we have not encountered any recurrent or metastatic tumor among the 18 bland or borderline looking cases, although follow-up was limited.

To date, 12 salivary neoplasms showing thymus-like phenotype have been reported under different names including carcinoma showing thymus-like elements (CASTLE), CASTLE tumor, intrasalivary thymic carcinoma, and ectopic thymic carcinoma [14–24]. However, two reported tumors likely have represented other entities including one EBV-positive lymphoepithelial carcinoma [16, 21]. Of the remaining 10 cases, 7 originated in the parotid, 2 in the submandibular, and 1 in the sublingual gland (Table 4). They affected seven females and three males aged 23 to 79 years (median, 55). Their size ranged from 1.3 to 5 cm (median, 2). A cystic component was noted in two cases (20%). Lymphovascular invasion and perineural invasion were recorded in three of six and two of five cases, respectively. Regional nodes were positive in six of nine cases (67%). One patient had a lung lesion suspicious for NSCLC, so that the precise site of the primary remains ambiguous. Based on either frankly malignant cytology, presence of necrosis, lymphovascular invasion, perineural invasion, positive regional nodes, and/or a combination thereof, all nine reported tumors with detailed information qualified as malignant. Notably, in none of these reported cases was a NSLA-like or benign component described.

**Table 4:** Clinicopathological features of reported thymus-like salivary gland neoplasms (*n* = 10)

No	ID	Age/sex	Site	Size (cm)	Cystic	Histological pattern	MI	LVI/Pni	Necrosis	Treatment	Nodal status	Outcome
1	Wong et al. [14]	55/F	PG	1.5	No	Basaloid elongated cells	NA	−/−	-	TPE + ND + aRT	+	NED (> 12 months)
2	Lorenz et al. [15]	79/F	PG	1.7	No	Solid basaloid elongated cells	NA	NA	NA	Surgery, ICI mets	+	Possible NSCLC same histology ICI and remission
3	Ardighieri et al. [17]	35/M	SLG	1.3	No	Focal squamoid nests HC-like abrupt keratin, ectopic thymus in neck	2	+/+	NA	Excision + ND + aRT	+	NED (24 months)
4	Ishikawa et al. [18]	23/F	PG	5	No	Focal squamoid nests HC-like abrupt keratin	NS	−/−	NA	SPE + ND + aRT	+	NED (13 months)
5	Yamamoto et al. [19]	31/F	PG	3.5	No	Basaloid, cytologically malignant	9	+/?	+	TPE + ND + aRT	−	NED (20 months)
6	Yamamoto et al. [19]	40/F	SMG	1.7	+	Basaloid, cytologically malignant	12	-/-	+	TE + ND	−	LN (4 months), lungs (50 months), rib (60 months), AWD (65 months)
7	Kunc et al. [20]	69/F	PG	3.8	No	HC-like foci	NA	NA	NA	RPE + ND + aRT	+	NED (12 months)
8	Uchiyama et al. [22]	58/M	PG	2	No	Basaloid elongated cells	NA	+/+	+	PE + ND + aRCT	−	NED (13 months)
9	Sasaki et al. [24]	56/F	SMG	NA	NA	NA	NA	NA	NA	NA	NA	NA
10	Hamada et al. [23]	63/M	PG	2.3	+	SCC-like with lymphocytes	NA	NA	NA	TPE + ND + aRT	+	NED (6 months)

*aRT* adjuvant radiation therapy, *MI* mitotic index, *LN* lymph node, *LVI* lymphovascular invasion, *NA* not available, *ND* neck dissection, *NED *no evidence of disease, *PG* parotid gland, *Pni* perineural invasion, *RPE* radical parotidectomy, *SLG* sublingual gland, *SMG* submandibular gland, *TPE* total parotidectomy

There are three possible explanations for the discrepancy regarding the frequency of malignancy in reported cases compared to our study. First, our study reevaluated all consecutive cases in our files and reclassified tumors in the spectrum of NSLA, irrespective of their biological behavior. We found that most of these tumors are histologically and clinically indolent or benign, though long-term follow-up was not available for most cases. Second, case reports are significantly biased toward malignant cases due to clinical interest in reporting them. Third, some of the reported cases originated in non-parotid sites and some were evidently other specific entities (e.g., EBV + lymphoepithelial carcinoma) unrelated to thymus-type neoplasms, limiting the reliability of these case reports in addressing the overall biology of these tumors. Taken together, our study indicates that the majority of lymphoepithelial salivary neoplasms with thymus-like phenotypes are benign or indolent lesions that may rarely undergo transformation into carcinoma with variable thymus-like features.

Regarding their defining immunomarkers, it is still premature to consider the mere expression of CD5 and CD117 in a salivary neoplasm enough to define a thymus-like phenotype. Notably, CD117 is frequently expressed in ductal and myoepithelial cells in a variety of benign and malignant salivary gland neoplasms. Saskia et al. investigated the expression of CD5 in a large cohort, encompassing most of the salivary gland tumor categories. They found no expression in any of the examined cases, highlighting the relative specificity of CD5 to thymus-like neoplasms [24]. However, two previously reported tumors that are morphologically and/or etiologically not thymus-compatible expressed CD5 and CD117, indicating the need to incorporate both exclusion and inclusion criteria when considering the possibility of a thymus-like phenotype in a salivary neoplasm [16, 21]. EBV status is mandatory to rule out lymphoepithelial carcinoma [7, 8].

The genetic landscape of these tumors has not been studied. The very few cases with molecular data showed no recurrent alterations [14, 18, 20]. In our current study, it was remarkable that four of seven tumors (57%) showed inactivating *CLYD* mutations.

*CYLD* mutation is a common driver of salivary neoplasms in the spectrum of membranous/spiradenoma-like basal cell adenoma and membranous basal cell adenocarcinoma, particularly in the context of Brooke-Spiegler syndrome [27, 28]. None of our cases had a history of Brooke-Spiegler syndrome or morphological features of membranous basal cell neoplasms. Lack of morphological features of membranous basal cell neoplasms in our cases points to the existence of a distinct *CYLD-*mutated salivary neoplasm with lymphadenoma-like morphology and thymic immunophenotype, hence expanding the family of *CYLD* mutant entities in the salivary glands.

Last but not least, the detected *YAP1::MAML2* gene fusion represents another interesting finding, linking these tumors with a thymus-like phenotype. Among epithelial neoplasia, this fusion characterizes the majority of metaplastic thymomas [29] and a subset of benign and malignant poroid neoplasms of skin adnexa [30] and rarely the salivary glands [31]. The tumor with this fusion was predominantly cystic with thymus-like epithelial components, lacking poroid features and did not have the morphological characteristics of cystic metaplastic thymoma [32].

Finally, p16 was frequently diffusely positive in these tumors, so that in conjunction with occasional cystic features, the possibility of cystic metastasis from HPV-associated oropharyngeal carcinoma can be raised. However, none of our cases was diagnosed with SCC before or after diagnosis of the thymus-like salivary tumor and the intraparotid location is exceedingly rare site of metastatic oropharyngeal carcinoma. Moreover, all cases tested for high-risk HPV were negative.

In summary, we herein describe detailed morphological, immunophenotypic, and genotypic characteristics of the largest series to date on thymus-like salivary neoplasms, highlighting a morphological continuum, rare occurrence of frankly malignant cases, probable relationship, and/or origin from non-sebaceous lymphadenomas and showing recurrent *CYLD* mutations in > 50% of cases. Recognition of more cases should help to delineate the full spectrum of these under-recognized lesions. Whether a classification scheme similar to their intrathyroidal thymic counterparts would be more reasonable remains to be addressed in the future.
